# Detection and Selection of Behavioral Patterns Using Theme: A Concrete Example in Grassroots Soccer

**DOI:** 10.3390/sports5010020

**Published:** 2017-03-13

**Authors:** Mario Amatria, Daniel Lapresa, Javier Arana, M. Teresa Anguera, Gudberg K. Jonsson

**Affiliations:** 1 Department of Physical Activity Sciences and Sports, Pontifical University of Salamanca, 37007 Salamanca, Spain; mamatriaji@upsa.es; 2 Department of Educational Sciences, University of La Rioja, 26004 Logroño, Spain; 3 Department of Education, International University of La Rioja (UNIR), 26006 Logroño, Spain; katrapuna@gmail.com; 4 Faculty of Psychology, University of Barcelona, 08035 Barcelona, Spain; mtanguera@gmail.com; 5 Human Behavior Laboratory, University of Iceland, 107 Reykjavík, Iceland; gjonsson@hi.is

**Keywords:** soccer, technical-tactical performance, T-patterns, theme, observational methodology

## Abstract

Observational methodology provides a rigorous yet flexible framework for capturing behaviors over time to allow for the performance of subsequent diachronic analyses of the data captured. Theme is a specialized software program that detects hidden temporal behavioral patterns (T-patterns) within data sets. It is increasingly being used to analyze performance in soccer and other sports. The aim of this study was to show how to select and interpret T-patterns generated by the application of three “quantitative” sort options in Theme and three “qualitative” filters established by the researchers. These will be used to investigate whether 7-a-side (F7) or 8-a-side (F8) soccer is best suited to the learning and skills development needs of 7- and 8-year-old male soccer players. The information contained in the T-patterns generated allowed us to characterize patterns of play in children in this age group. For both formats, we detected technical-tactical behaviors showing that children of this age have difficulty with first-touch actions and controlling the ball after a throw-in. We also found that ball control followed by a pass or a shot at the goal are common in the central corridor of the pitch. Further, depth of play is achieved by ball control, followed by dribbling and a pass or shot. In F8, we saw that depth of play was achieved through ball control, followed by dribbling and passing of one or more opponents leading to a pass or shot. However, in F7, we saw that players succeeded in advancing from their goal area to the rival goal area through a sequence of actions.

## 1. Introduction

Data acquisition, data mining, and context-aware analysis have become crucial areas of research in team ball games, such as soccer [1]. Game analysis is changing at a dizzying pace, with constant improvements to automatic recording and annotation systems that enable the immediate acquisition of large amounts of complex data [2]. The potential commercial applications of these systems are further driving the development of increasingly small and precise sensor modalities and tracking devices. These can provide valuable information for clubs that can afford to deploy this technology. Data on player positions and displacements, however, need to be enriched with other types of data if they are to truly improve our understanding of the game. Insights into how technical skills are applied to tactical game situations, for example, would be potentially relevant to most studies of this kind [3]. Although incipient efforts have been made to achieve fully automatic data acquisition [1], most data are still recorded semi-automatically via visualization, recording, and coding software [4].

Analysis of play is challenging in soccer, as soccer is a complex, many-sided product of dynamic interactions between rival players who execute actions underpinned by different yet complementary facets. These facets are related to numerous aspects such as skill, physique, and tactics [5,6]. Much of the research in soccer has sought to identify the different factors that contribute to performance [7,8]. To explain these factors, studies typically investigate associations between two or more variables (e.g., pitch area, defensive-offensive technical-tactical actions and moments of play) [9,10]. However, as valuable as such analyses may be, they reduce complex game situations to a single, synchronous value [11,12]. Soccer, however, is a complex game that cannot be understood through a simple analysis of frequencies. To truly grasp the complexities of the game, it is necessary to analyze patterns of behavior, which can be revealed by both the order (and hence frequency) of events and their duration (and hence order) [3,13].

Observational methodology [14] has gained increasing recognition in recent years as a rigorous yet flexible methodological framework [15] for capturing behaviors as they occur [16]. Thus permitting the subsequent diachronic analysis of data that leads to the detection of patterns of behavior [17,18,19]. Theme is a powerful algorithm-based software program that detects temporal patterns, known as T-patterns, hidden within sets of data [20,21]. The program has made a considerable mark in different academic fields since the launch of a free downloadable version in 2012 (www.patternvision.com).

As described by Magnusson [22], data sets in Theme are seen as a collection of n (>1) T-series—point series representing the occurrence of event types within an observation period [1, T], where T is the duration of the observation period corresponding to the number of data points (events) in the data set. The number of occurrences of an event type divided by T gives the average probability of this event type occurring in a given time unit, which is then used as the baseline probability by the detection algorithms. Theme searches for critical intervals [t + d1, t + d2](d2 ≥ d1 ≥ d0) that contain an occurrence of A followed by B that is higher than would be expected by chance according to a pre-established level of significance. To do this, it compares the null hypothesis that A and B are independently distributed and that B has a fixed probability of occurrence per unit of time (=NB/T) during the observation period (where N is the number of occurrences of B and T is the duration of the observation period). When Theme detects an occurrence of A followed by B within a critical interval, it generates a simple T-pattern (AB). Occurrences of simple T-patterns become events, which subsequently constitute focal event types at the next detection level. Theme repeats this process, moving up, level by level (from 1 to n), in search of critical interval relationships featuring the patterns detected in previous levels to generate increasingly complex T-patterns. 

The algorithm that searches for what are known as “fast” critical interval relationships uses the occurrence of A as the lower limit of the critical interval and starts the detection process from the greatest distance between A and B, i.e., [0, max(B − A)]. It tests the significance of each possible relationship by narrowing the search to the next shortest distance between A and B until it finds a significant critical interval relationship. If it fails to find one, it reaches what is known as a [0, 0] interval. The process for detecting free critical interval relationships differs in version 5 and 6 of Theme. In Theme 5, the software restricts its search between two extremes (longest and shortest distance between A and B), starting from the extreme with the lowest P value. In Theme 6, by contrast, it increases the lower limit of the critical interval until it finds a significant critical interval or reaches max (B − A, max (B − A)]. The free interval, as it is known in Theme 5, is called the free heuristic critical interval in Theme 6.

Theme has been used to analyze patterns in numerous sports [23,24,25], including soccer [26,27,28], which is at the forefront of research in sport.

Generally, when analyzing data sets of behaviors recorded with ad hoc observation instruments [29], they are semi-automated [4], Theme detects a series of T-patterns generated by more or less restrictive search parameters. This output then needs to be analyzed to select the T-patterns that are most relevant to the research question being investigated. Large numbers of T-patterns are common when working with large samples, even when very strict search parameters are applied [30]. This can cause confusion among researchers, who may be tempted to focus on patterns with the highest number of events (groups of clusters). There are, however, many options for searching and choosing T-patterns, including automatic default sort options in Theme and qualitative filters established by the researcher, according to the aims of the study [31]. In this study, we take a real-life example from the field of soccer analysis to show how different research questions can be answered in Theme, with a particular focus on the processes involved in detecting and selecting T-patterns. Our primary objective, which is methodological in nature, is to show how to process T-patterns detected by Theme and vary search parameters to produce results that reflect different aspects of the study question. To our knowledge, this has not been done in previous studies.

To achieve this primary objective, we use a real-life example consisting of analyzing different game formats used in youth soccer in a regional soccer league in Spain. The relationship between the number of players and pitch size influences the physical, technical, and tactical performance of young soccer players [32,33]. National soccer federations across Europe recommend using game formats that are suited to the development and learning needs of children of different ages as they move up to 11-a-side soccer. Competition formats, however, can vary considerably from region to region [34]. Our example is framed with a real-life situation in which a regional soccer federation in Spain was deliberating on whether children moving up from futsal (5-a-side format played under identical conditions to adult futsal) would benefit more from a 7-a-side (F7) or 8-a-side (F8) competition format in terms of the acquisition of technical and tactical skills. The secondary objective of this study, thus, which was substantive in nature, was to analyze the technical-tactical performance of 7- and 8-year old children in F7 and F8 to determine which format was most appropriate to their development and learning needs.

## 2. Method

We employed an observational methodology design [14], which, according to the designs described by Anguera, Blanco-Villaseñor, Hernández-Mendo, and Losada [35], can be classified as nomothetic, follow-up, and multidimensional. It is nomothetic because the players observed were from different teams. Follow-up was inter- and intra-sessional as we performed a frame-by-frame analysis of six F7 and F8 soccer matches played by 7- and 8-year-old boys during a triangular tournament; this exhaustive study permitted the subsequent detection of T-patterns underlying the children’s behavior [36]. The multidimensional nature of the design was determined by the multiple dimensions in the observation instrument, which corresponded to proxemic or gestural behaviors. Observation was non-participative (the observers did not participate in the matches) and the degree of perceptivity was total (direct observation of video recordings).

### 2.1. Participants

The research project was approved by a scientific committee at the University of La Rioja in accordance with the Ethical Principles of Psychologists and Code of Conduct of the American Psychological Association, and the rules of the Ethics Committee of the Spanish Association of Psychologists.

We used a convenient sample [37] consisting of players from the three top-ranking teams in a regional Spanish soccer league at the end of the season. To collect the study data, we organized a triangular tournament in which each team played the other two teams using both the F7 and F8 formats. Each team had nine or ten players, born in 2003, who were 7 or 8 years old at the time of the tournament. In the preceding season, they had all played futsal, which is the competition format used for children of this age, and they were all due to move up to either F7 or F8. None of the players had prior experience with F7 or F8, thereby meeting the requirements for inter-sessional consistency. The players and trainers who participated in the tournament were the same as those who had participated in the league during the previous season. The only tactical indication given to trainers was that they had to use a 1-2-3-1 formation in F7 and a 1-3-3-1 formation in F8. 

Given the influence of the size of the game area and the number of players in the offensive phase of soccer [38,39] the matches were all played on an artificial pitch size (65 m × 45 m) with the reglementary number of players and using a no. 4 ball. A rest period of 15 min was left between matches. Each match consisted of two 15-min halves, with a half-time period of five minutes. All the matches were refereed by the same referee.

### 2.2. Observation Instrument

The observation instrument used to code the data was built from three references sources: (a) The Football Observation System (SOF) (version 4) [40]; (b) pitch divisions as proposed by Arana, Lapresa, Anguera, and Garzón [41]; and recommendations for the development of ball skills for children aged 6–10 years [42].

The instrument was a combination of a field format system and category systems. The field format system comprised different dimensions, each of which formed the basis for an exhaustive and mutually exclusive category system (Table 1).

### 2.3. Recording and Coding

Six matches (three F7 and three F8 matches) were video recorded during the tournament. All the minutes of play (30 per match) were analyzed. This analysis produced six data sets: one for each attacking team in each match. In total, 90 min of play were recorded per format. The sample consisted of 324 sequences of actions and 947 contacts for F7, and 346 sequences of actions and 953 contacts for F8. 

The data were analyzed and coded using the LINCE software program (version 1.2.1) [43]. The video recordings were focused on the movement of the ball, i.e., the camera followed the player with the ball at all times.

### 2.4. Data Reliability

The reliability of the data was ensured using a consultative agreement method [44], involving two observers. The first observer coded all the data on two occasions. The second observer then searched for discrepancies between matching pairs of data and made a final decision on which option to include in the definitive data set. All the discrepancies identified were due to incorrect alignment due to differences in the identification of a sequence of actions [45]. Cohen's kappa statistics were calculated using GSEQ (version 5.1) [46]. The kappa statistic for each of the six data sets (match-team observed) for each game format was higher than 0.80 in all cases, indicating “almost perfect” agreement according to the criteria of Landis and Koch [47].

### 2.5. Data Analysis

The full data set for each game format (F7 and F8) was analyzed to identify offensive patterns of play. This resulted in the detection of T-patterns that would not have been detected had the individual data sets been analyzed.

The following search parameters were set in Theme (for more information, see the reference manual [48]): (a) frequency of occurrence of ≥3; (b) significance level of 0.005 (0.5% probability of critical interval being due to chance); (c) redundancy reduction setting of 90% (exclusion of T-patterns when >90% of occurrences of a new pattern start and finish with the same critical interval relationships of patterns already detected); (d) deactivation of fast requirement at all levels and selection of free heuristic critical interval setting; (e) validation of results through randomization of data on five occasions, with acceptance only of patterns for which the probability of the randomized data coinciding with the real data is 0; (f) application of simulation filter in Theme (version 5.0), which performs randomizations according to the established significance level. In our case, 2000 randomizations (1/0.005 × 10) were performed. This means that any T-pattern detected is added to the output if Theme detects it, among all the randomly generated relationships, (n/2000) < 0.005 critical interval relationships with interval intervals of the same size as or smaller than those of the relationship being tested.

## 3. Results

Six dimensions, 51 categories, and 358 event types were detected in the F7 data set. There were 1789 events, which corresponds to a mean frequency of 4.99 events for each event type. In total, 184 T-patterns met the search criteria. There were 145 patterns with two events, 38 with three events, and just one with four events. In the data set for the F8 matches, we identified six dimensions or criteria, 52 categories, and 364 event types. There were 1865 events, which corresponds to a mean of occurrence of 5.12 events per event type. A total of 203 T-patterns met the search criteria; 160 featured two events, 42 three events, and one with four events.

The next challenge was to select relevant T-patterns for subsequent analyses. To this end, we used three “quantitative” filters, available in Theme, and three “qualitative” filters chosen to suit the objective of our study.

**Figure 1:**
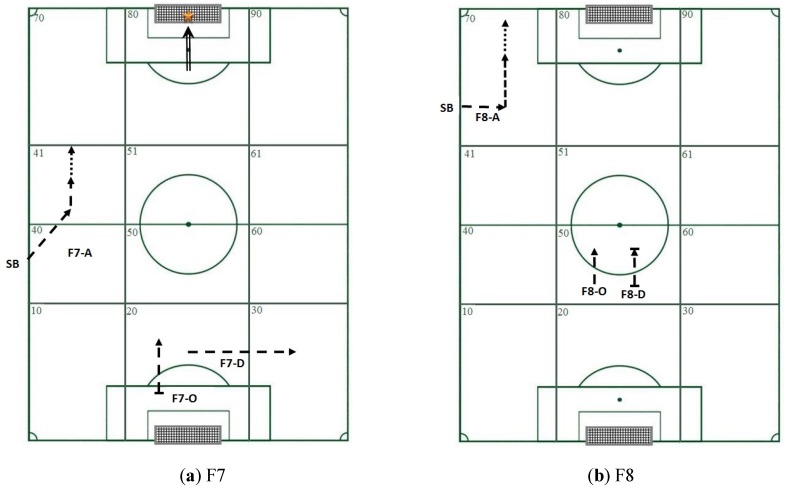
Diagram showing the information contained in the T-patterns selected using the sort settings in Theme (greatest length -A-, greatest frequency -O- greatest duration –D-): TI = Throw-in, 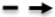 = single touch (C1), 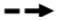 = attempt to control the ball with 2 or more touches resulting in loss of ball (C12), 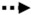 = Occasional interception with continuation of play, 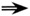 = Shot, 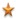 = Goal, 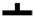 = Recovery of ball, 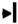 = Loss of ball. (**a**) F7, (**b**) F8.

The three default settings used to sort the patterns were length (patterns with the highest number of events), frequency (patterns with the highest number of occurrences), and duration (patterns with the longest duration between the first and last event in the pattern) (See Table 2 and Figure 1).

Use of the above sort options frequently generates an overwhelmingly long list of patterns and it may be tempting to choose the patterns at the top of the list. In our opinion, this is where the researcher’s work truly starts, i.e., it is here that they need to choose the automatic sort option that will produce the information most suited to the qualitative filters that will be selected to answer the research question. The aim of our study was to analyze technical and tactical behaviors by 7- and 8-year-old soccer players in two game formats, F7 and F8, to investigate which format is best suited to the learning needs of children at this age.

**Figure 2:**
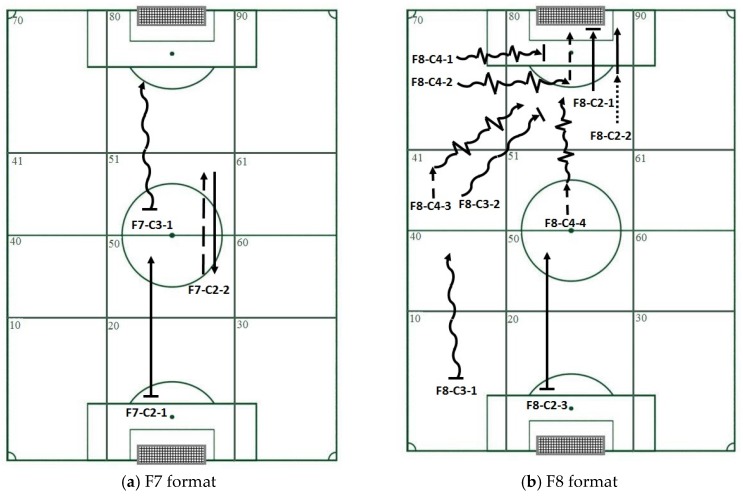
Diagram showing the information in the T-patterns selected to reflect ball contacts recommended for skills development in young soccer players: 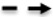 = single touch (C1), 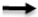 = control of the ball followed by a pass or a shot at the goal (C2), 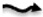 = control of ball, followed by dribbling and a pass or shot (C3), 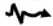 = control of ball, followed by dribbling and passing of one or more opponents, and a pass or shot (C4), 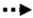 = Occasional interception with continuation of play, 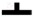 = Recovery of ball, 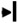 = Loss of ball. (**a**) F7, (**b**) F8.

Considering that the observation instrument was specifically built to analyze technical and tactical actions, the first step was to select T-patterns including types of ball contact recommended by experts for the skills development of players of the ages analyzed (Table 3 and Figure 2). These consisted of the most frequent T-patterns that included events C2 (control of the ball followed by a pass or a shot at the goal), C3 (control of ball, followed by dribbling and a pass or shot), or C4 (control of ball, followed by dribbling and passing of one or more opponents, and a pass or shot) (Table 3). There were seven or more occurrences of T-patterns featuring C2. The maximum number of occurrences was seven in F8 and eleven in F7. The maximum number of T-pattern occurrences containing C3 was four for F8 and six for F7. Finally, a maximum of three T-patterns featuring C4 were detected for F8. C4 was not present in any of the patterns detected for F7. 

The second qualitative filter applied was designed to analyze sequences of actions starting in the goal area of the team being observed (zone 20) and ending in the zone containing the rival goal (zone 80). This information reflects the ability to move the ball up the pitch towards the rival goal. The output generated by Theme using the default sort settings contained one T-pattern for F7 and none for F8 (Table 3).

**Figure 3:**
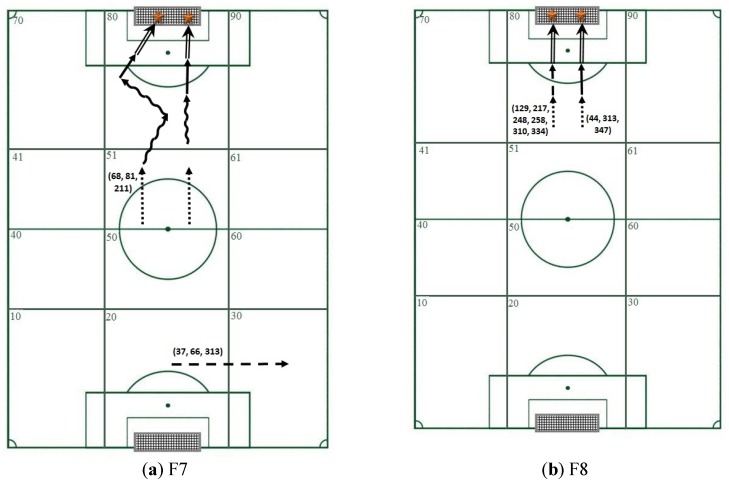
Diagram showing sequences based on information contained in T-patterns detected using qualitative search options to reflect the scoring of a goal. No. = sequences in which T-patterns are detected, 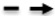 = single touch (C1), 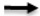 = control of the ball followed by a pass or a shot at the goal (C2), 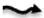 = control of ball, followed by dribbling and a pass or shot (C3), 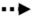 = Occasional interception with continuation of play, 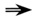 = Shot and 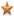 = Goal. (**a**) F7, (**b**) F8.

The third filter was used to analyze sequences of actions ending in a goal, which is obviously one of the most interesting aspects of soccer [49]. Table 4 and Figure 3 show the T-patterns containing the event goal generated by Theme following application of the search settings. This last option additionally shows how researchers can address aspects inherent to the issue of degradation [50] by using information contained within patterns to create more complex game sequences of events (sequences of action sequences) by combining different T-patterns that occur within the same sequences of actions. These can be identified by the number showing the order in which they occur within the sample [51]. Table 4, for example, shows that the patterns featuring the goal cluster detected for F7 can be grouped into two packets of sequences of action sequences: 68, 81, and 211 (underlined in Table 4) and 37, 66, and 313 (shown in italics in Table 4). A similar situation was detected for F8, for which two packets of sequences of action sequences were also detected: 129, 217, 248, 258, 310, and 334 (underlined in Table 4) and 44, 313, and 347 (shown in italics). Analysis of the internal intervals, i.e., the time interval between adjacent events in the pattern, can reveal additional information on these important sequences leading up to a goal.

## 4. Discussion

Apart from providing a robust means of observing and collecting data in natural settings, observational methodology provides a rigorous yet flexible framework for capturing behaviors that occur over a period of time that can then be subject to diachronic analysis [15]. The use of Theme to conduct diachronic analyses of behavior in soccer and other sports is growing [52]. There are several explanations for the increasing popularity of this tool. First, it is an extremely powerful means of detecting behavioral patterns that remain invisible to the naked eye [30]. Second, the groups of clusters that make up T-patterns are highly informative as they show concurrent, prospective, and retrospective relationships between events. Third, the launch of a free version of Theme for academic use (Theme.edu) in 2012 has made this tool accessible to researchers.

The analytical power of Theme, however, means that the program may generate an overwhelming number of patterns that, if not processed adequately, can cause confusion. The aim of this study was to use a real-life example to show how the use of quantitative (automatic) filters in Theme and qualitative filters chosen by the research team can facilitate the selection of T-patterns that can shed light on the research question (Table 5).

Of the patterns detected using the three default sort options in Theme, we chose the patterns at the top of the list to illustrate how these settings can help to narrow the possibilities for answering the research question. Obviously, T-patterns with the largest number of events are the most “evident” patterns and it may be tempting to automatically choose these due to the amount of information they contain. The patterns detected using the sort by length setting are typically related to patterns detected using the sort by level setting, which orders patterns according to the number of hierarchical levels they contain [50]. These two settings are related, as the more events in a pattern, the more complex the resulting tree structure will be in terms of relationships between simple T-patterns or between T-patterns and satellite clusters that are connected by a critical interval relationship within the broader pattern. 

The main advantage of the sort by frequency setting is that it identifies the most common T-patterns in the data set. A disadvantage, however, is that patterns at the top of the list generally contain two events and as such are not very informative. For our study, we applied this filter after first applying a qualitative filter to select patterns containing events C2, C3, and C4 (contacts with the ball). Use of this filter had a double aim: to produce information relevant to our research question and to illustrate how T-patterns can be narrowed down.

The filter used to order T-patterns by duration (patterns with the longest duration between the first and last event in the pattern) has the advantage that it enables the selection of T-patterns that cover most of the sample, but it has two important disadvantages. Firstly, clusters tend to be separated from each other in time and secondly, the patterns include clusters (rows in the data set) that generally have other interspersed events from the data set that are not reflected in the T-pattern. Note that deactivation of the fast requirement in Theme tends to increase the time between the clusters that form a T-pattern.

We will discuss our use of the three qualitative filters applied to help answer our research question, i.e., whether F7 or F8 was more suited to the learning and skills development needs of 7- and 8-year old soccer players. The first, logical, step was to select all T-patterns including information on contact with the ball. In our case, we searched for C2 (control of the ball followed by a pass or a shot at the goal), C3 (control of ball, followed by dribbling and a pass or shot), and C4 (control of ball, followed by dribbling and passing of one or more opponents, and a pass or shot). These moves were included in the SOF observation tool as they represent skills that according to the literature in this field should be taught in children of this age [42,53,54].

The second filter was applied to detect T-patterns containing clusters showing sequences of actions in which the attacking team succeeded in advancing the ball from zone 20 (own goal area) to zone 80 (rival goal area). Studies have highlighted the difficulty of reaching the rival goal area from the other end of the pitch [41,55]. As shown by Castellano [56], as the team moves closer to the rival goal, the retrospective perspective becomes less defined while the prospective perspective becomes more defined.

The aim of the third filter applied was to identify T-patterns containing a cluster leading to a goal; these T-patterns therefore correspond to sequences of actions which resulted in the most important offensive action in a soccer match, a goal [57]. 

The above approach shows how the use of different filters can shed light on different aspects of the same situation, providing complementary insights that can contribute to a greater overall understanding. In the next section, we will show how the results generated by the different filters helped us to answer our research question. 

The length filter (number of events in a pattern) produced patterns consisting of a throw-in from the sideline, followed by a single contact with the ball in both F7 and F8. These results highlight the difficulties that children have controlling the ball in this situation in both game formats; this difficulty has also been detected in U12 soccer players [58]. Application of the frequency filter, which shows how often a pattern occurs, showed that the rival team recovered the ball after a single touch by an attacker, which, considering the difficulty that children of this age have with ball control, suggests that one-touch actions are not recommended [59,60,61]. Finally, the duration filter, which shows the total duration of pattern occurrences, ordered from longest to shortest, shows how the patterns generated for both F7 and F8 were affected by a loss of information as they were missing events that formed part of the sequence but not of the pattern. In F7, the T-pattern selected using this filter coincides with the pattern selected using two of the qualitative filters chosen by the researchers: goal and depth of play. The relationship is coherent due to the time it takes to cover the full length of the pitch in a single sequence. Therefore, this pattern can be considered as an indicator of quality of play in F7 [62]. The T-pattern obtained for F8 reflects a sequence consisting of loss and recovery of the ball following a single contact.

With respect to the qualitative filters chosen specifically for the purpose of the study, we will first focus on interpreting the information in the T-patterns generated in terms of recommended ball contacts for children of this age [33,54,63]. T-patterns featuring C2 (control + pass or shot) were detected in the central corridor in both F7 and F8. Patterns showing recovery of the ball in zone 20 were also seen in both game formats, followed by movement of the ball to the next zone in the central corridor through C2 (which could correspond to a throw-in by the goalkeeper). The T-patterns containing C3 (control of ball, followed by dribbling and a pass or shot) detected in F7 and F8 reflect the achievement of a greater depth of play, represented by the movement of the ball from one sector to the next in all cases. T-patterns incorporating C4 (control of ball, followed by dribbling and passing of one or more opponents, and a pass or shot) were detected only in F8. The T-patterns detected show sequences in which players, using this form of contact, achieved both greater depth of play (movement of ball from a sector further back in the pitch to a more advanced sector) and greater width of play (reflected only in patterns starting in the left corridor (zone 41 and zone 70) with progression to the central corridor in zone 80, i.e., the zone containing the rival goal).

Our analysis of generated T-patterns that reflect offensive sequences starting in the safety sector (area containing the attacking team’s goal) and ending in the definition sector (area containing the defending team’s goal) revealed a single T-pattern in F7 (which was the same as that generated using Theme's sort by duration setting) and no patterns in F8. This finding lends support to the idea that the larger number of players and greater defensive balance in F8 may make it more difficult to build an attack than in F7 [60,64].

Finally, we analyzed all the T-patterns detected using the pre-established filter settings that reflected the scoring of a goal in some of their clusters. These were represented graphically to allow us to relate different T-patterns that occurred in the same sequences of actions based on the order of the game sequences in the sample [46]. In both F7 and F8, the game sequences reflected by these T-patterns show that goals are scored from shots taken in zone 80 (the rival goal area). The tendency to score goals from the area immediately surrounding the goal is logically consistent with the nature of the game [57,65] but it is even more prevalent at young ages due to the difficulty of reaching the goal from greater distances [34]. The game sequences for F7 show progression from the definition sector through the creation sector in the rival’s half of the pitch, while for F8, all the events took place in zone 80.

The information revealed by the T-patterns generated using the different applied search filters [66] shows how researchers can investigate different aspects of the same situation. In our case, we saw, for both F7 and F8, that (a) 7- and 8-year-old male soccer players have difficulty with first-touch actions and controlling the ball following a throw-in from the sideline; (b) C2 (control of the ball followed by a pass or a shot at the goal) is used repeatedly in the central corridor; and (c) goals are scored from the goal area. In F8, C4 (control of ball, followed by dribbling and passing of one or more opponents, and a pass or shot) achieved depth of play (progression of ball to a sector further up the pitch) and width of play (preferentially from the left to the right corridor). Finally, we found that F7 offered players more opportunities than F8 to advance the ball from their goal area to the rival goal area.

With regard to the substantive aim of our study, and in line with previous reports that simplified game formats such as F7 and F8 are better suited to the physical and technical-tactical needs of young soccer players [30,67], our findings show that both F7 and F8 are appropriate formats for children moving up from futsal. The results from our comparison of F7 and F8 are thus consistent with reports in the literature and lend support to arguments made in favor of both game formats [68]. The fact that children have greater difficulty in moving the ball up the pitch in F8 than F7 is probably due to the greater tactical balance, both between and within lines, associated with the 1-3-3-1 formation in F8. The F7 formation, 1-2-3-1, by contrast, creates a greater imbalance, making it easier for players to create spaces. This imbalance could favor the acquisition of technical-tactical skills in children of this age [60,69]. 

Our study has practical applications related to both our objectives. From a methodological perspective, our example shows how T-pattern analysis can be used to detect behavioral patterns that are reflected in real-life situations. In addition, it provides researchers or other interested parties with a guide on the steps involved in detecting and selecting relevant T-patterns in Theme. An important point in this regard is that all the software programs used in this study are freely available. Our findings also have substantive applications as they provide relevant information on the technical-tactical performance of 7- and 8-year-old children in F7 and F8.

Finally, the design of our study represents a step forward in overcoming the limitations typically associated with observational studies of performance in sport. Our intention is to continue to harness advances in automatic tracking and data analysis systems [1], while at the same time enriching the resulting data and searching for meaningful patterns that will help to build a viable theoretical framework [70] that integrates contextual information with performance-related data such as technical, tactical, and physiological characteristics.

## Figures and Tables

**Table 1:** Observation instrument.

No.	Dimensions	Categories (Codes)
1	Ball possession	PO) Possession of ball by team being observed; PC) Possession of ball by opposing team; Inob) Unobservable
2	Action initiation zone	ZS10, ZS20, ZS30–safety sector; ZS40, ZS50, ZS60–creation sector; ZS70, ZS80, ZS90–definition sector (see Figure 1, Figure 2 and Figure 3)
3	Action conclusion zone	ZE10, ZE20, ZE30–safety sector; ZE40, ZE50, ZE60–creation section; ZE70, ZE80, ZE90–definition sector.
4	Contact with ball	C1) single touch and regulatory throw-in/kick-in; C12) attempt to control the ball with 2 or more touches resulting in loss of ball; C2) control of ball (including picking up of ball by goalkeeper) followed by a pass or shot -regardless of whether the ball reaches a team member or is recovered by an opponent; C23) control of ball, followed by dribbling, and loss of ball; C24) control of ball, followed by dribbling, attempt to go around one or more opponents, and loss of ball; C3) control of ball, followed by dribbling and a pass or shot–regardless of whether the ball reaches a team member or is recovered by an opponent; C4) control of ball, followed by dribbling and passing of one or more opponents, and a pass or shot–regardless of whether the ball reaches a team member or is recovered by an opponent; C5) Header.
5	Interruptions	FDFT) free kick for team being observed; FDFJ) offside for team being observed; FFSB) throw-in for team being observed; FFSE) corner kick for team being observed; FFSP) goal kick for team being observed; CDFT) free kick against team being observed; CDFJ) offside against team being observed; CFFB) throw-in against team being observed; CFFF) corner kick or goal kick against team being observed; GF) goal scored by team being observed; GC) goal conceded by team being observed; SN) neutral kick.
6	Interceptions	P) loss of ball; R) recovery of ball; IOC) Occasional interception with continuation of play.
7	Shot	TG) shot resulting in goal; TI) shot intercepted by opponent other than the goalkeeper; TM) shot between the posts not resulting in a goal; TF) shot wide of the posts; TP) shot saved or cleared by the goalkeeper.

**Table 2:** T-patterns detected using automatic sort options in Theme (quantitative filters).

Setting	Identifier	String-Like Pattern	Occurrences/Length/Duration (in Frames)	Mean Internal Interval (in Frames)
Length	F7-A	((zi40,zf40,ffsb zi40,zf41,c1)	4/4/311	1.25/54.00/21.50
		(zi41,zf41,c1 zi41,zf41,ioc))		
	F8-A	((zi70,zf70,ffsb zi70,zf70,c1)	3/4/350	4.00/76.67/35.00
		(zi70,zf70,c12 zi70,zf70,ioc))		
Frequency	F7-O	(zi20,zf20,r zi20,zf20,c1)	14/2/116	7.29
	F8-O	(zi50,zf50,r zi50,zf50,c1)	15/2/883	57.87
Duration	F7-D	(zi20,zf30,c1 (zi80,zf80,tg zi80,zf80,gf))	3/3/1389	441.00/21.00
	F8-D	(zi50,zf50,r (zi50,zf50,c1 zi50,zf50,p))	7/3/938	70.86/62.14

**Table 3:** T-pattern detected using qualitative search options to reflect contacts—control of the ball followed by a pass or a shot at the goal (C2), control of ball, followed by dribbling and a pass or shot (C3), control of ball, followed by dribbling and passing of one or more opponents, and a pass or shot (C4)—and depth of play (sequences of actions starting in the goal area of the team being observed—zone 20—and ending in the area containing the rival goal—zone 80) (first and second qualitative filters).

Search Option	Game Format-Identifier	String-Like Pattern	Occurrences/Length/Duration (in Frames)	Mean Internal Interval (in Frames)
C2	F7-C2-1	(zi20,zf20,r zi20,zf50,c2)	11/2/391	34.55
C2	F7-C2-2	(zi50,zf51,c1 zi51,zf50,c2)	10/2/131	12.10
C2	F8-C2-1	(zi80,zf80,c2 zi80,zf80,p)	7/2/392	55.00
C2	F8-C2-2	(zi80,zf80,ioc zi80,zf80,c2)	7/2/311	43.43
C2	F8-C2-3	(zi20,zf20,r zi20,zf50,c2)	7/2/68	8.71
C3	F7-C3-1	(zi51,zf51,r zi51,zf80,c3)	6/2/199	32.17
C3	F8-C3-1	(zi10,zf10,r zi10,zf40,c3)	4/2/561	1.50
C3	F8-C3-2	(zi41,zf80,c3 zi80,zf80,p)	4/2/561	139.25
C4	F7	None detected		
C4	F8-C4-1	(zi70,zf80,c4 zi80,zf80,p)	3/2/370	122.33
C4	F8-C4-2	(zi70,zf80,c4 zi80,zf80,c1)	3/2/340	112.33
C4	F8-C4-3	(zi41,zf41,c1 zi41,zf80,c4)	3/2/86	27.67
C4	F8-C4-4	(zi51,zf51,c1 zi51,zf80,c4)	3/2/148	48.33
Depth	F7-D	(zi20,zf30,c1 (zi80,zf80,tg zi80,zf80,gf))	3/3/1389	441.00 / 21.00
	F8	None detected		

**Table 4:** T-patterns detected using qualitative search options to reflect the scoring of a goal. The T-patterns in both formats correspond to two different packets of game sequences (underlined and italics) (third qualitative filter).

Format	String-Like Pattern	Occurr/Length/Dur	Order in Sequences of Actions	Mean II
F7	(zi51,zf80,c3 (zi80,zf80,tg zi80,zf80,gf))	3/3/490	68, 81, 211	136.33/26.00
F7	(zi80,zf80,c3 zi80,zf80,gf)	3/2/303	*37*, 105, 211	100
F7	(zi80,zf80,c2 (zi80,zf80,tg zi80,zf80,gf))	3/3/224	68, 207, *313*	54.67/19.00
F7	(zi20,zf30,c1 (zi80,zf80,tg zi80,zf80,gf))	3/3/1389	*37, 66, 313*	441.00/21.00
F7	(zi51,zf51,ioc ( zi80,zf80,tg zi80,zf80,gf))	3/3/610	68, 81, *313*	177.00/25.33
F8	(zi80,zf80,c1 ( zi80,zf80,tg zi80,zf80,gf))	6/3/140	129, 217, 248, 258, 310, 334	1.17/21.17
F8	(zi80,zf80,ioc ( zi80,zf80,tg zi80,zf80,gf))	3/3/162	129, *313*, 334	34.33/18.67
F8	(zi80,zf80,c2 ( zi80,zf80,tg zi80,zf80,gf))	3/3/114	*44, 313, 347*	21.67/15.33

**Table 5:** Quantitative (default sort settings in Theme) and qualitative (search options chosen by researchers) filters used to select T-patterns (the options used in this study are shown in italics).

Quantitative Search Options	Qualitative Search Options
*Frequency*	*Ball contacts*
*Length*	Combination of contacts
Level	Combination of contacts according to pitch area
Actors	*Sequences of play that end in a goal*
Actor switches	Sequences of play that include a shot
First occurrence time	*Sequences of play that start in zone 20 and end in zone 80*
*Total duration*	Sequences of play that start in the safety sector and end in the definition sector
Mean p in pattern (mean statistical significance value for the critical intervals in a pattern)	Sequences of play involving the two side corridors
Median p in pattern	Sequences of play involving the two lateral corridors before reaching zone 80
Maximum p in pattern	Sequences of play that include strategic situations: corners , penalties, free kicks
Different event-types	Combination of contacts and tactical situations (combination of above)
Different items	etc.
